# Molecular and clinical characterization of autoimmune polyendocrinopathy-candidiasis-ectodermal dystrophy syndrome (APECED) in Iranian non-Jewish patients: report of two novel AIRE gene pathogenic variants

**DOI:** 10.1186/s13023-021-02170-z

**Published:** 2022-01-06

**Authors:** Aria Setoodeh, Samareh Panjeh-Shahi, Fariba Bahmani, Fatemeh Vand-Rajabpour, Nazanin Jalilian, Fatemeh Sayarifard, Farzaneh Abbasi, Azadeh Sayarifard, Parastoo Rostami, Nima Parvaneh, Haleh Akhavan-Niaki, Mohamadreza Ahmadifard, Mina Tabrizi

**Affiliations:** 1 Division of Endocrinology and Metabolism, Growth and Development Research Center, Children’s Medical Center, Tehran University of Medical Sciences, Tehran, Iran; 2 Cellular and Molecular Biology Research Center, Health Research Institute, Babol University of Medical Sciences, Babol, Iran; 3 Department of Medical Genetics, School of Medicine, Tehran University of Medical Sciences, Tehran, Iran; 4 Department of Clinical Biochemistry, School of Medicine, Kermanshah University of Medical Sciences, Kermanshah, Iran; 5 Growth and Development Research Center, Children’s Medical Center, Tehran University of Medical Sciences, Tehran, Iran; 6 Division of Allergy and Clinical Immunology, Department of Pediatrics, Children’s Medical Center, Tehran University of Medical Sciences, Tehran, Iran; 7 Department of Medical Genetics, Faculty of Medicine, Babol University of Medical Sciences, Babol, Iran

**Keywords:** APECED, APS1, Autoimmunity, *AIRE*, Addison, Candidiasis

## Abstract

**Objective:**

Autoimmune polyendocrinopathy-candidiasis-ectodermal dystrophy syndrome (APECED) is a rare autosomal recessive systemic autoimmune disease caused by mutations in the autoimmune regulator (*AIRE)* gene. Incidence of this genetic disorder is estimated at 1/90,000–200,000 worldwide and 1/6500–9000 in genetically isolated populations such as Iran. Here, we investigated *AIRE* gene mutations in eight independent Iranian non-Jewish families.

**Methods:**

We sequenced the coding regions of the *AIRE* gene and documented mutations which were further confirmed in respective parents.

**Results:**

In total, 11 cases from 8 independent families were recruited. Mucosal candidiasis, Addison’s disease and hypoparathyroidism were the most common clinical manifestations in these patients. One novel homozygous splice acceptor mutation (c.308-1G>C), and one novel heterozygous stop-gain mutation (c.1496delC) combined with a known heterozygous c.232T>C missense mutation were found. Moreover, we observed previously described splice donor (c.1095+2T>A), frameshift (c.967-979del), stop-gain (c.415C>T), and missense (c.62C>T) mutations among the patients. All results were co-segregated in parents.

**Conclusion:**

Here, we reported two novel mutations in the *AIRE* gene leading to APECED. Our data could provide insight into the phenotypic and genotypic spectrum of APECED in the non-Jewish Iranian population. These findings, in addition to future functional assays, can elucidate disease-causing mechanisms related to the AIRE gene and assist in genetic counseling and diagnosis.

## Introduction

Autoimmune polyendocrinopathy-candidiasis-ectodermal dystrophy syndrome (APECED) [MIM 240300], also known as autoimmune polyglandular syndrome type 1 (APS-1), is a rare autosomal-recessive systemic autoimmune disease often appearing early in life, typically in infants at 3–5 years of age [1]. APECED is known, to date, as an organ-specific human autoimmune disorder affecting several organs and is described to have a Mendelian inheritance pattern. The disease is reported worldwide and occurs in about 1 in 90,000–200,000 people in most populations [2]; however, the lifetime incidence of APECED is exceptionally high in genetically isolated populations such as Iranian Jews [3] with a calculated prevalence of 1/6500–1/9000 based on published documents [4].

This monogenic disorder results from mutations in the autoimmune regulator (*AIRE*) gene assigned to chromosome 21q22.3 [5]. The genomic structure of the *AIRE* gene, approximately 13 kb in length, consists of 14 exons, encoding a polypeptide of 545 amino acids [6]. The gene encodes a transcriptional regulator, constructs nuclear bodies, and interacts with the transcriptional coactivator cAMP-response element-binding protein (CREB). Presence of a conserved nuclear localization signal (NLS) at the N terminus followed by a SAND domain and four LXXLL motifs are the most substantial characteristics of the AIRE protein [7]. Recent studies have revealed the key role of the encoded protein in shaping central immunological immunity [8]. *AIRE* is predominantly expressed in the medullary thymic epithelial cells (mTECs); however, to some extent, it is also expressed in rare hematopoietic cells of lymph nodes. AIRE protein provokes expression of a wide array of tissue-restricted proteins in mTECs presented on class I and II major histocompatibility complexes (MHCs) to select non-autoreactive T cells through thymic media. The “projection of self” process, mediated by mTECs, is critical for deletion of autoreactive T cells and for development of the main phase in prevention of autoimmunity [9].

Several mutations in the *AIRE* gene correlate with development of organ-specific autoimmune diseases with a monogenic autosomal recessive inheritance pattern. Among these mutations, the R257X nonsense mutation is common in the Finnish and Eastern European population [10], the Y85C missense mutation has high incidence in isolated populations such as Finns (1:25,000 individuals) [11] and Iranian Jews (1:9000) [12], as well as the R139X nonsense mutation in Sardinian patients (1:14,500) [13]. In addition, a 13-bp deletion, located in exon eight (1085–1097), is ubiquitous and can be routinely found in Norwegians, British, and North American populations [14].

Interestingly, signs and symptoms of APECED can widely vary among affected individuals and populations. Presence of primary adrenocortical failure, hypoparathyroidism, and chronic mucocutaneous candidiasis are the most common clinical symptoms for APECED [15]. However, the clinical phenotype can be described as a highly variable combination of autoimmune reactions towards different endocrine and non-endocrine organs [16]. Therefore, researchers believe that differences in clinical manifestations of this pathology might be attributed to the various specific *AIRE* gene mutations. This hypothesis can emphasize the importance of mutational analysis of the *AIRE* gene as one of the most important diagnostic tools for early detection of this life-threatening syndrome and the subsequent approach to its treatment [13]. To date, several studies have investigated the frequency of different *AIRE* gene mutations in Iranian Jews, but there are few studies of *AIRE* gene mutations among patients with APECED in non-Jewish Iranians [17]. Therefore, the primary aim of this study was to screen the coding regions of the *AIRE* gene in Iranian patients to determine possible mutations involved in incidence of this syndrome.

## Materials and methods

### Families

Eight non-Jewish Iranian families, consisting of eleven affected individuals together with their healthy family members, were recruited among patients with APECED admitted to the Children’s Medical Center from January to September 2017. In three out of eight families, two affected individuals were studied, and the other five families had only one affected individual in the pedigree. Informed written consent was obtained from all participants and the study was approved by the Institutional Review Board (IRB), with Ethics Committee approval code of IR.MUBABOL.HRI.REC.1397.149.

### Diagnostic criteria and clinical data

The diagnostic criterion was presence of at least two of the following major disease components clearly documented by a physician: hypoparathyroidism (subnormal Ca^2+^ and supranormal inorganic phosphate concentrations in serum, excluding renal failure), primary adrenocortical failure (primary cortisol and/or aldosterone deficiency), and chronic mucocutaneous candidiasis (CMC) [18].

Typical biochemical findings in combination with bone density tests and electrocardiogram (EKG) were used for diagnosis of hypoparathyroidism. Abnormal tooth development was also evaluated in children by an experienced dentist. To confirm adrenal failure, results of the ACTH stimulation tests were interpreted by endocrinologists. To assess the adrenal gland stress response, adrenal response to the adrenocorticotropic hormone was measured. Finally, possible associated hypothyroidism was diagnosed using the thyroid function tests. Liver function was also performed in order to rule out the rare possibility of autoimmune hepatitis. Diagnosis of chronic mucocutaneous candidiasis was made in the occasion of recurrent skin or mucosal candidiasis in absence of diabetes and antibiotic use.

### Primer design

Referenced and designed primer sequences of the *AIRE* gene (NM_000383) were extracted from the National Center for Biotechnology Information (NCBI) database (Table 1). Then, primers were blasted against the human genome using Primer-BLAST and any possible secondary structures of the designed primers were predicted using Gene Runner software version 3.01.

**Table 1:** Primer sequences used for amplification of exons and exon—intron boundaries of the *AIRE* gene

Exon	Forward primer	Reverse primer
1	CTTTGCTCTTTGCGTGGTCG	GACTATCCCTGGCTCACAG
2	CCTGGGAGCTCCACCCTCTAGT	CACCACTCCGGTTCCAAGTCCA
3	TGGCCAAGGTGTCCAGTTCT	TCTAGTACCCAGAGGAGACC
4	GCAAAGGGACTACCCAGCACT	TAGGACAGGGTCTCAGAGGGCA
5	GCTGCCTGCTTCTGGCATAGA	GGCGTGGTCCTCCTTCCATCT T
6	TCTGCTAGACCCCACCCTG	GCCCCCAGCAGAGCCACT
7	GAACAGCGTTGCCTCTGG	AGTGCCCAGGTAAAGGCA G
8	GGAGTTCAGGTACCCAGAGA	TGACTCAGAACCCCTTTCCA
9	CTGGGGTTTGGGGATCTGTC	GGGACATAGTGCTATGGCTGG
10	CCACTCAGTGTGGACGCCTT	TGAATTCATCCGCCCCGTAG
11	GTGAGGCTCCTCACTTGCGCCTAG	TGTGGTTGTGGGCTGTATGATGTG
12	CACACCCCATACCCCGGA	CTGGTGCAAGCCCTCGAAG
13	CCTGCGGCCTCTGTACCC	AGAGTGGGGAGCCTGGGTG
14	AGGTTCTCACCGTCACTCTGT	ACTGACAAGAGGTGGCGCTGT

### DNA extraction and PCR amplification

Genomic DNA was extracted from 5 ml of patient EDTA-treated blood samples using the standard DNA salting out method [19]. Subsequently, all 14 exons of the *AIRE* gene and their flanking exon–intron boundaries were investigated in the patients with APECED. In brief, 200 ng of genomic DNA (gDNA) was amplified via a 35-cycle PCR in which the initial 10-min denaturation at 95 °C was followed by a denaturation at 95 °C for 30 s, annealing for 30 s, and final extension at 72 °C for 30 s, in 25 μl of total volume containing 10 × Buffer solution, 0.2 mm of each dNTP, 2.5 mm MgCl_2_, 0.4 μm of each primer, and 2 U of Taq Gold DNA Polymerase (Applied Biosystems, USA). PCR amplifications were performed with specific primers for each exon in a GeneAmp® PCR system 9700 thermal cycler (Applied Biosystems, USA). The PCR products were sequenced by means of a direct chain termination method using BigDye Terminator v1.1 Cycle Sequencing Kits (Applied Biosystems, USA). The sequence products were then purified using the Microcon columns (Millipore) and analyzed on an ABI PRISM 310 automated DNA sequencer (Applied Biosystems, USA).

### Validation of the variants and in silico analysis

Sequence variant numbering was based on the transcript ENST00000291582.6 for *AIRE*. All novel variants were named according to the guidelines of the Human Genome Variation Society (http://www.hgvs.org/). Upon finding a variant, the following methodology, which is based on the ACMG’s guideline for interpretation of sequence variants [20], was adopted in order to validate each variation:

First, segregation analysis was performed for variants in the whole family. In case of novel variants, fifty healthy controls of the same ethnicity were screened along with the patients using Sanger sequencing. Next, an extensive in silico study was performed for the variant.

An extensive search was conducted through databases Ensemble.org, dbSNP (http://www.ncbi.hlm.nih.gov/snp), 1000 genome databases (http://browser.1000genome.org), HGMD (www.hgmd.cf.ac.uk), Iranome (http://www.iranome.ir), ExAc browser (exac.broadinstitute.org), gnomAD (https://gnomad.broadinstitute.org/), and recently published articles in PubMed.

Possible pathogenic effects of the novel variants were checked by mutation taster (http://www.mutationtaster.org/) and CADD algorithms (https://cadd.gs.washington.edu/snv). In the CADD database, variants with scores more than 14 are categorized as pathogenic.

In addition, BDGP (http://www.fruitfly.org/seq_tools/splice.html), Human Splicing finder (http://www.umd.be/HSF3/) and NetGen2 (http://www.cbs.dtu.dk/services/NetGene2/) were used in order to predict possible effects of splice site mutations.

## Results

### Clinicopathological features

**Fig. 1:**
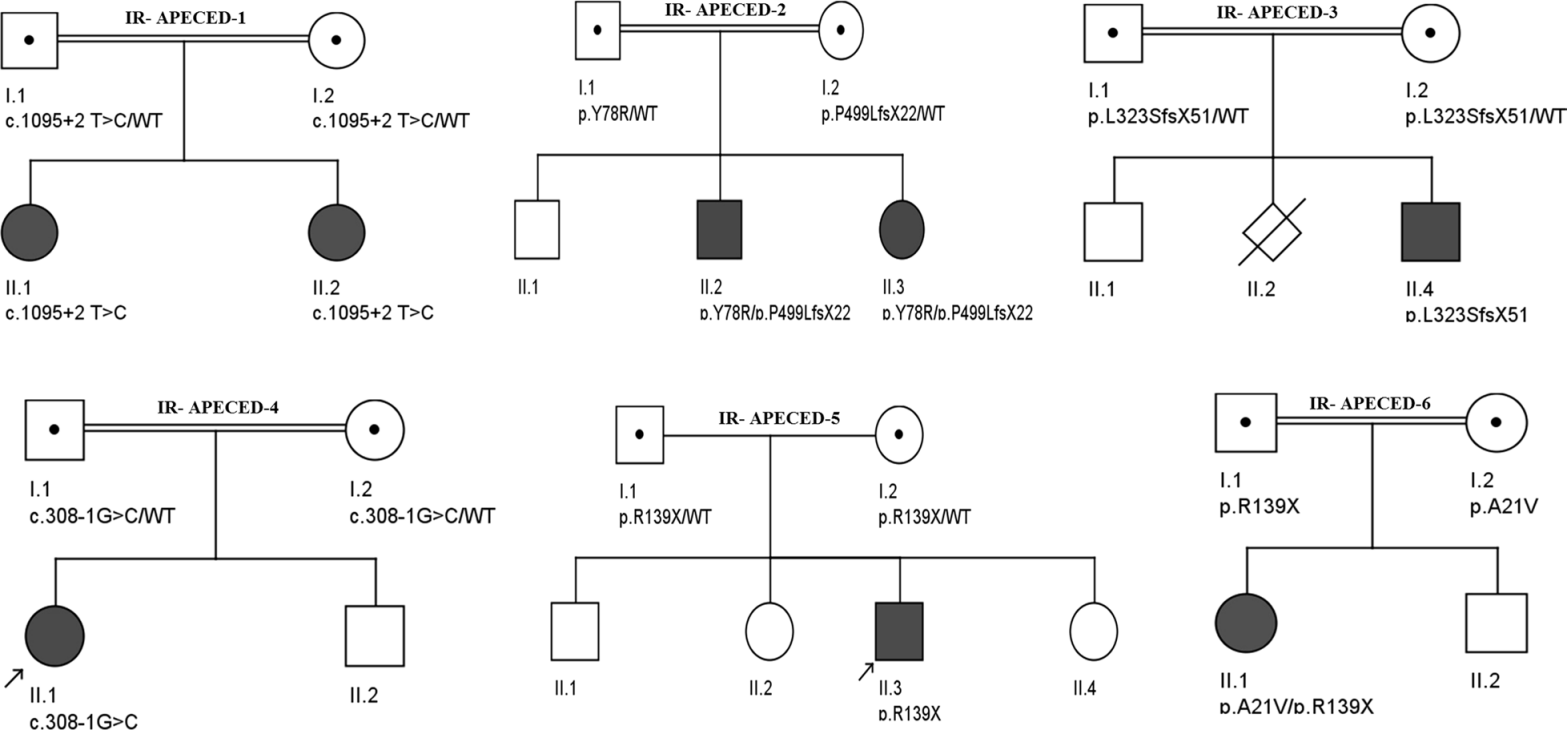
Pedigrees of families APECED1-6. Circles—female subjects; squares—male subjects; open circle and open square—normal; filled circle and filled square—affected

In total, eleven cases including 7 women and 4 men from 8 unrelated APECED families along with their healthy family members were recruited. The ages ranged from 2 to 26 years and consanguinity was noted in 6 out of the 8 pedigrees. Participant pedigrees and clinical characteristics are depicted in Fig. 1 and Table 2, respectively. The initial and the most frequently observed manifestation of APECED was mucosal candidiasis (CMC) which presented mostly at one year of age, followed by Addison’s disease and hypoparathyroidism. On the other hand, vitiligo, chronic malnutrition, autoimmune hepatitis and enamel hypoplasia were the least frequent symptoms detected only once in these families. Detailed information regarding frequency of major symptoms and their distribution among cases are presented in Table 2.

**Table 2:** Clinical manifestations in patients with autoimmune polyendocrinopathy-candidiasis-ectodermal dystrophy syndrome (APECED)

Families	Patients	Sex	Age	Age of onset	Consanguinity	Mucosal candidiasis	Hypoparathyroidism	Addison disease	Autoimmune thyroiditis	Insulin dependent-diabetes mellitus	Keratitis	Nail dystrophy	Additional findings
IR-APECED-1	Patient 1	F	15	CMC = 1 Y; Hypo = 4 Y	+ First cousin	+	+	+	** − **	** − **	+	+	
	Patient 2	F	12	CMC = 2 Y; Hypo = 4 Y		+	+	+	** − **	** − **	+		Vitiligo
IR-APECED-2	Patient-3	M	12	CMC = 13 M; Hypo = 4.5 Y; AD = 4.5 Y	+ First cousin	+	+	+	+	+	−		
	Patient 4	F	10	CMC = 14 M		+	+	+	−	−	−		
IR-APECED-3	Patient 5	M	15	CMC = 1 Y; HYPO = 4 Y	+ First cousin	+	+	+	+	−	−		Chronic malnutrition, Autoimmune hepatitis
IR-APECED-4	Patient 6	F	26	CMC = 1 Y; HYPO = 7 Y; AD = 7 Y	+ First cousin	+	+	+	−	−	−	+	
IR-APECED-5	Patient 7	M	20	CMC = 1 Y; HYPO = 3 Y; AD = 5 Y	−	+	+	−	−	−	+	+	
IR-APECED-6	Patient 8	F	13	< 1 Y	+	+	+	+	−	−	−	−	
IR-APECED-7	Patient 9	M	10	CMC = 1 Y; AD = 8 Y	+ First cousin	+	+	+	+	+	−	+	Enamel hypoplasia
	Patient 10	F	2	CMC = 10 M; AD = 6 M		+	−	+	−	−	−	+	Enamel hypoplasia
IR-APECED-8	Patient 11	F	14	5 Y	−	+	+	+	−	−	−	−	
		7 F/4 M		Total		11/11	10/11	10/11	3/11	2/11	3/11	5/11	

### *AIRE* gene mutations

**Fig. 2:**
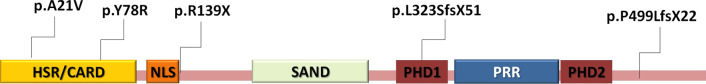
APECED structure and localization of mutations reported in this study

In this study, four homozygote and two compound heterozygote pathogenic variants were detected in 6 out of the 8 families studied. These variations included 5 previously reported (c.232T>C, c.967_979delCTGTCCCCTCCGC, c.1095+2T>A, c.415C>T and c.62C>T) and two apparently novel variants (c.1496delC and c.308-1G>C). A summary of detected mutations is provided in Table 3 and the location of the mutations at protein level is depicted in Fig. 2. Results of in silico analysis of the splice site variants found in this study are presented in Table 4.

**Table 3:** Identified mutations in the *AIRE* gene from non-Jewish Iranian patients

Families	Mutations Detected	Variant effect	Functional Consequence	Position	Genotype	Reference
IR-APECED-1	c.1095+2T>A	Splice donor loss	Splice donor variant	IVS9	Hom	Seifi-Alan
IR-APECED-2	**c.1496delC**	**p.P499LfsX22**	**Stop gained**	**Exon 12**	**Compound heterozygote**	**This study**
	c.232T>C	p.Y78R	Missense	Exon 2		Cihakova [12]
IR-APECED-3	c.967_979delCTGTCCCCTCCGC	p.L323SfsX51	Frameshift variant	Exon 8	Hom	Nagamine [5]
IR-APECED-4	**c.308-1G>C**	**Splice acceptor loss**	**Splice acceptor variant**	**IVS2**	**Hom**	This study
IR-APECED-5	c.415C>T	p.R139X	Stop gained	Exon 3	Homo	Rosatelli [13]
IR-APECED-6	c.415C>T	p.R139X	Stop gained	Exon 3	Compound heterozygote	Rosatelli [13]
	c.62C>T	p.A21V	Missense	Exon 1		Halonen [30]

Novel variants identified in this study are shown in bold

**Table 4:** Results of in silico analysis of the splice site variants found in this study

Variant	Mutation taster	Human splice finder	BDGP	NetGen2	CADD C-score^a^
c.308-1G>C	Disease causing	Broken WT Acceptor Site	WT = 0.85; MUT = NR	WT = 0.99; MUT = NR	26.2
c.1095+2T>A	Disease causing	Broken WT Donor Site	WT = 0.97; MUT = NR	WT = 0.95; MUT = NR	23.0

^a^A score of greater or equal 20 indicates the 1% most deleterious and so on

The First novel pathogenic variant, c.1496delC, was observed in IR-APECED-2. In this family, a compound heterozygous mutation, consisting of one novel heterozygous stop-gain mutation (c.1496delC) and a known heterozygous c.232T>C missense mutation were observed in both affected offsprings. Interestingly, their first cousin parents were heterozygous for c.232T>C (Y78R) and c.1496delC (P499LfsX22) variations, respectively (Fig. 1). The former led to substitution of Trp codon with Arg at codon 78, and the latter deletion would change the amino acid sequence, subsequently causing a premature stop codon. Eventually, both of these heterozygous variations in the gene resulted in malfunctioning of the AIRE protein.

Another novel variant found in our study came from family IR-APECED-4 (Fig. 1). As shown in the pedigree, a homozygous splice acceptor variant (c.308-1G>C) was discovered in the patient (subject II:1) and parents illustrated heterozygous genotypes.

Of note, by sequencing the coding regions of the *AIRE* gene in patient families Nos. 7 and 8, no mutation was found (families not shown in the pedigree).

## Discussion

Pathogenic variants in the *AIRE* gene cause APECED, which is described as autoimmune damage to the endocrine glands and multiple ectodermal dystrophies. The chronic or recurrent mucocutaneous candidiasis completes the APECED clinical picture [21]. Studies suggest that the protein encoded by the *AIRE *gene plays a critical role in regulating certain aspects of the immune system function. Therefore, mutations in the *AIRE* gene can reduce or eliminate function of the autoimmune regulator protein and will subsequently cause APECED [22]. Though mutations in *AIRE* are not common in many countries, this condition occurs more frequently in certain populations, affecting approximately 1 in 9000–25,000 individuals among Iranian Jews, Sardinians, and Finns.

By sequencing the complete coding region as well as the exon–intron boundaries of the *AIRE* gene, we found four homozygous and two compound heterozygote mutations in six out of eight pedigrees with APECED and the heterozygous forms were further confirmed in their parents. Except for c.1095+2T>A (intron 9), R139X (exon 3) and p.L323SfsX51 (exon 8) (pathogenic mutations in the *AIRE* gene that have been previously reported in the Iranian non-Jewish population [17, 23]), this study demonstrated, for the first time, other disease-causing mutations (Table 3) in non-Jewish Iranian families. This could be of particular importance for clinicians and patients with relevant phenotypes to consider the possibility of a wide range of APECED mutations in different ethnicities in regions of the Middle East such as Iran .

**Fig. 3:**
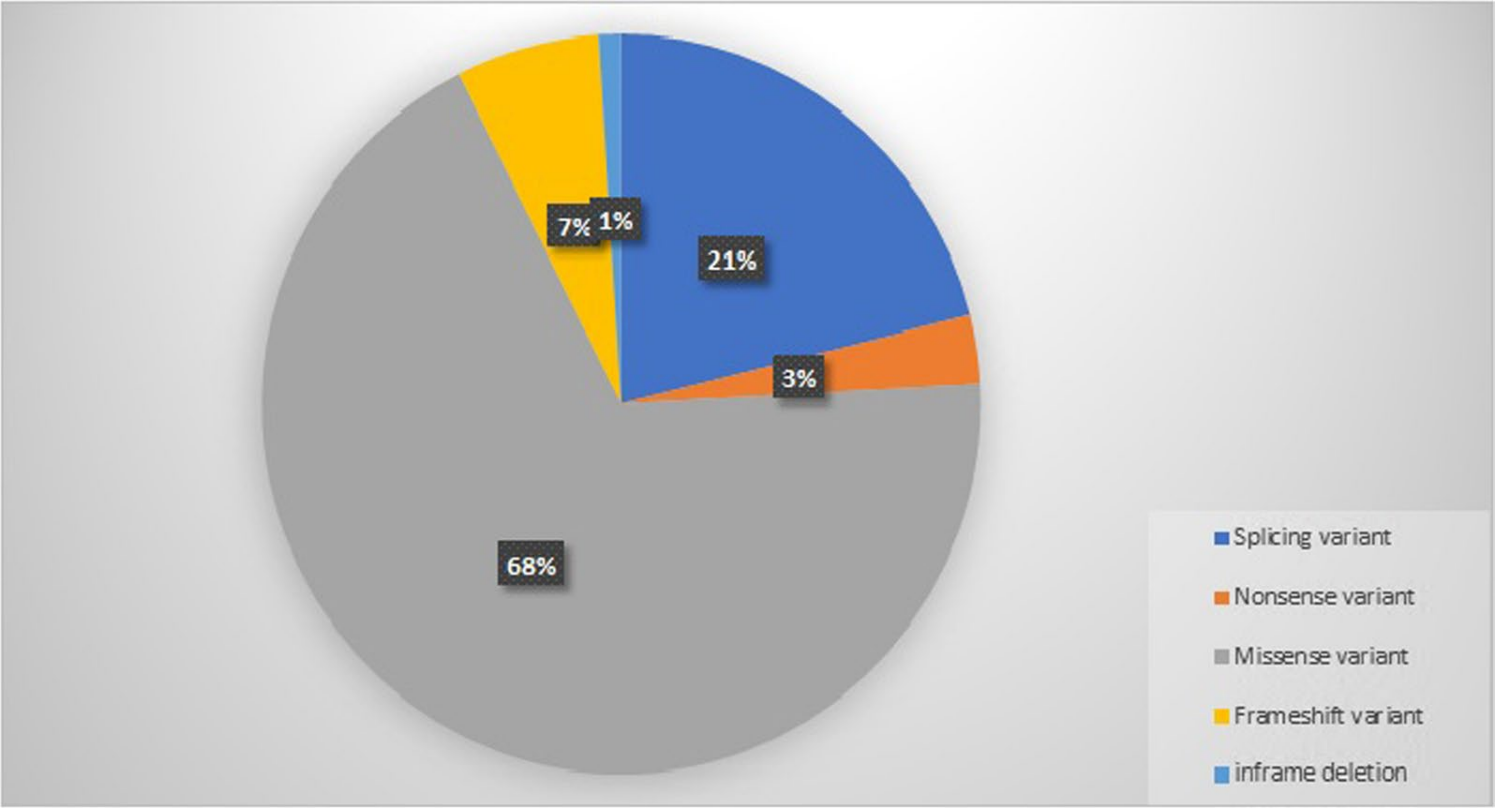
Graph representing distribution of *AIRE* mutations based on mutation type

The variants we reported here were almost equally distributed among categories of missense (2 of 7), stop-gain (2 of 7), splicing (2 of 7) and frameshift (1 of 7) variants. However, statistically analyzing the total number of variants extracted from ensemble.org demonstrated that missense variants are the most frequent variants within the AIRE gene, comprising 68% of variants, followed by splice site variants (Fig. 3).

Clinically, CMC, Addison’s disease and hypoparathyroidism were the most frequent findings in our study population. These manifestations are also the most penetrant phenotypes reported in similar studies. On the other hand, here we demonstrated the complete triad in 10 out of 11 cases. This rate seems to be greater than the average percentage reported in the literature (up to 63%). In accordance with our findings, Fardi Golyan et al. [23] reported a frequency of 80% for the classic triad in their Iranian APECED cases. Therefore, simultaneous presence of the three APECED cardinal signs can be regarded as a unique finding in the Iranian non-Jewish APECED patients. This finding needs to be further evaluated in larger sample sizes and populations.

In contrast to Iranian Jewish patients that rarely have mucocutaneous candidiasis, all of our non-Jewish patients, surprisingly, had mucocutaneous candidiasis. Researchers believe that differences in the effects of specific *AIRE* gene mutations, as well as variations in other genes that have not yet been identified, may help explain why the signs and symptoms of APECED can vary among affected individuals and populations. Thus, study of isolated populations, such as Iranian Jews, added to extended inbred pedigrees are useful aids for delineating genotype–phenotype correlations as well as mapping genes and genetic variations explaining the phenotypic heterogeneity of the disease [24]. However, findings in isolated populations might not be always valid in the general population [3]. In such cases, fine mapping in the general population, rather than focusing on genetically isolated populations, can be considered an alternative strategy for identification of genetic aberrations in rare monogenic disorders with Mendelian inheritance such as APECED.

In some cases, our results were in line with previous findings in other populations [3, 25]; nonetheless, occurrence of several new nucleotide variations as novel changes in the *AIRE* gene was also detected among our patients. For instance, results of recent studies in the Finnish population revealed that the C to T substitution at nucleotide 889 located in exon six is the most common mutation worldwide [26]. This mutation changes CGA into TGA as a stop codon and subsequently leads to a truncated 256 residue protein. This is found in about 85% of patients with APECED with a carrier frequency of 1:250 in randomly selected unaffected Finns [26]. Nonetheless, this mutation is not a rare polymorphism and, interestingly, it was not found in non-Jewish Iranian patients of this study.

A 13-bp deletion in exon eight has been found in a number of patients with APECED worldwide and is the second most common mutation in Finnish patients with APECED [27]. Based on results obtained in this study, this mutation has occurred in one out of eight non-Jewish Iranian patients in homozygous form. This deletion occurs in the region encoding the first PHD finger (Fig. 2), resulting in a protein with no intact PHD finger [28].

Two out of the seven pathogenic variants observed here resided in splice sites. The novel pathogenic variant c.308-1G>C was located in the second intron of the *AIRE* gene. This splice acceptor variant represented a disease-causing score of 26.2 in the CADD algorithm. The c.1095+2T>A substitution was a previously reported splice donor variant. This splice site change was predicted by in silico analysis to affect all downstream motifs in the protein including LXXLL and PHD domains. Also, by the CADD algorithm, this variation is predicted to be deleterious (score of 22.3). Interestingly, this variant was first identified in an Iranian APECED pedigree [17] and to the best of our knowledge has not been reported elsewhere so far. Thus, this variant can be subjected to further analysis as a putative founder mutation in the Iranian non-Jewish population.

Furthermore, in our patient series, we observed two homozygous missense mutations in the *AIRE* gene. These mutations are of particular interest because they may reveal functionally critical regions of the APECED protein. One of the mutations was c.232T>C substitution in exon 2, identified in family No. 2, and has been previously reported to be Pathogenic/Likely Pathogenic in APECED 1. Substitution of c.62C>T in the affected child of family No. 6 was the other missense mutation in our study. These single nucleotide exchanges have disease-causing prediction in both MutationTaster and CADD databases and are predicted to severely affect the HSR domain. This domain, is also present in the amino terminus of the human and mouse Sp100 and Sp140 proteins, is predicted to possess a function in regulation of gene expression and has recently been demonstrated to act as a homo-dimerization domain as well as being responsible for targeting these mentioned proteins to nuclear bodies. This dimerization might be crucial for DNA-binding or for binding to a currently unknown partner, and consequently, these mutations could disrupt such interactions. This clustering of missense mutations in the HSR domain provided further evidence for functional significance in the APECED protein [29, 30].

No evidence was found to prove any correlation between mutation type or mutation location with the clinical symptoms observed in our patients. Therefore, applying functional analysis of the APECED protein both in vitro and in animal models may elucidate mechanisms underlying molecular pathogenesis of this disease.

## Conclusion

We identified two novel mutations and five other previously reported mutations (other ethnicities) in eight Iranian non-Jewish patients with APECED. No mutation was found in three other cases which may be suggestive of other loci being involved in pathogenesis of APECED in the Iranian non-Jewish population. Identification of new genetic variations in the coding region of the *AIRE* gene associated with APECED might facilitate genetic diagnosis, prognosis, and potential treatment of the disease. Moreover, these findings may further elucidate mechanisms underlying autoimmune disorders such as APECED.

## Data Availability

Supporting information are available from the corresponding author upon request.
